# Location Optimization of Urban Fire Stations Considering the Backup Coverage

**DOI:** 10.3390/ijerph20010627

**Published:** 2022-12-29

**Authors:** Liufeng Tao, Yuqiong Cui, Yongyang Xu, Zhanlong Chen, Han Guo, Bo Huang, Zhong Xie

**Affiliations:** 1 School of Computer Science, China University of Geosciences, Wuhan 430074, China; 2 State Key Laboratory of Geo-Information Engineering, Xi’an 710000, China; 3 National Engineering Research Center of Geographic Information System, China University of Geosciences, Wuhan 430074, China; 4 Guangdong Hong Kong Macau Joint Laboratory for Smart Cities, Shenzhen 518034, China; 5 Key Laboratory of Urban Land Resources Monitoring and Simulation, Ministry of Natural Resources, Shenzhen 518034, China; 6 Wuhan Zondy Cyber Science and Technology Co., Ltd., Wuhan 430073, China

**Keywords:** location optimization, fire station, backup coverage model, maximal coverage model

## Abstract

Urban fires threaten the economic stability and safety of urban residents. Therefore, the limited number of fire stations should cover as many places as possible. Moreover, places with high fire risk should be covered by more fire stations. To optimize the location of urban fire stations, we construct a multi-objective optimization model for fire station planning based on the backup coverage model. The improved value of environment and ecosystem (SAVEE) model is introduced to quantify the spatial heterogeneity of urban fires. The main city zone of Wuhan is used as the study area to validate the proposed method. The results show that, considering the existing fire stations (85 facilities), the proposed model achieves a significant 38.56% in high-risk areas that can be covered by more than one fire station. If the existing fire stations are not considered when building 95 fire stations, the proposed model can achieve coverage of 50.07% in high-risk areas by utilizing more than one fire station. As a result, the proposed backup coverage model would perform better if the protection of high-risk areas is improved with as few fire stations as possible to guarantee more places covered.

## 1. Introduction

Fires can be a great threat to humans, infrastructure, and nature, and can often lead to loss of life, economic losses, and pollution [1]. The demand for fire station backup services is essential in cities with higher fire risk, these areas often tend to have a heavy workload in terms of fire and reduced quality of service. Moreover, to save costs, the limited number of fire stations should cover as more places as possible [2]. The placement of fire stations for easy response and recovery is a relevant issue faced today. 

Many towns and cities have an insufficient number of fire stations, such that the service scope of a single fire station is extensive. Experts and scholars have established a variety of optimization models and algorithms for fire station site selection to address these problems. The location set coverage model (LSCP) was developed in the 1970s to identify the location of emergency facilities [3]. The maximum coverage model, which mainly calculates how demand points can be covered with the least facilities, was subsequently proposed based on the LSCP [4]. When location optimization models for fire stations were built, the type of points used improved from binary [5] to continuous possibilities [6]. However, fire-fighting service areas with different fire risks require different degrees of fire station coverage [7]. Several models have been proposed to determine reasonable areas of responsibility and alleviate problems associated with the vagueness of decision-makers in terms of fire station location optimization [8,9]. However, these methods fail in situations in which demand points are not sufficient to provide fire protection when multiple fire accidents simultaneously occur in the same service area. Therefore, these methods cannot be used to optimize the location of urban fire stations in areas with high fire risk, which require more coverage. For example, areas with more than one point of demand in high fire risk areas or more than one high fire risk area is covered by the same emergency service facility; these scenarios are likely to suffer from simultaneous major emergency events. In such a case, a lack of fire service stations to provide emergency protection could lead to considerable damages. Moreover, several other constraint conditions should also be considered, including: the criteria that the distribution of urban fire stations should follow urban fire planning principles; limiting driving distances in terms of time and free-flow speed; and building more fire stations in areas with high fire risk.

To reduce the harm caused by fire and optimize the location of urban fire stations, two issues need to be resolved: (1) How can potential fire risk points be estimated? (2) How can a multi-objective optimization model be built that can select fire stations locations that cater to both high-risk areas that require more fire stations for sufficient coverage and a limited number of fire stations to cover as many places as possible? Additionally, an effective location selection model should resolve the demand for fire stations, but also pay attention to not exceeding the safety limits for each fire station [10]. Although several methods have been developed considering spatial partitioning and service area delimitation problems, establishing a spatial proximity model that addresses the issues mentioned above is a challenging task.

Recently, large volumes of geospatial data have emerged [11,12,13,14,15,16] that can be used to estimate potential fire risk points. Points of interest (POIs), effective and easily obtained geospatial data, have been widely used for urban computations [17,18]. These data represent real geographical entities, such as fire stations, hospitals, schools, railway stations, and supermarkets, in the form of spatial location points. POI data can also be used as a quantitative risk proxy for different functional spaces [19,20,21], and have been used to measure fire risk [22,23,24]. Several weighted sum methods, such as the analytic hierarchy process [25,26], expert scoring method [24], and information entropy method [27], have been used to quantify risk factors using POIs. However, these methods are insufficient as they involve subjective weighting evaluation and cannot describe the spatial heterogeneity of fire points. In this study, to improve the process of quantifying fire risk and retain the spatial features of POIs, an improved spatial approximate and value of environment and ecosystem (SAVEE) model based on grids [28] was utilized. 

In this study, POI data were used to quantify the spatial fire risk using the grid SAVEE method. The SAVEE grid method considers spatial relationships to calculate the risk at a particular fire point. A backup coverage model was established that comprehensively considers multiple constraint conditions so that high fire risk areas are covered by multiple fire stations and the limited number of fire stations can cover as many places as possible. Comparing the performance of the maximum coverage model with the proposed backup coverage model demonstrated good performance in high-risk areas. The results act as a reference for decision-makers in government agencies regarding the location of fire stations. 

## 2. Methods

The proposed backup coverage model is a multi-objective optimization model of candidate fire stations. Firstly, according to the spatial distribution of six types of POI fire impact factors, the density of each impact factor of each grid is calculated by using kernel density analysis. Based on the grid SAVEE model, the density measurement results of different factors are standardized and superimposed, and the total fire risk values of each grid can be obtained. Then, according to the grid size and the service range of the fire station, all the candidate points in the research area are obtained by the PIPS. As the requirement of the specific space distributions of the fire station, the candidate points are preliminarily screened. Finally, based on the principle of the fire station’s location, the optimization objectives and constraints required for the optimal deployment of the fire station are obtained.

### 2.1. Calculation of Urban Fire Risk Based on SAVEE

#### 2.1.1. Fire Risk Assessment Based on POIs

POIs represent real geographical entities representing spatial location points, such as fire stations, hospitals, schools, train stations, and supermarkets. The fire-related risk POI classification method used in this paper is mainly derived from previous research. Firstly, the POIs affecting fire risk are divided into regional fire risk, vulnerability, and disaster resistance [22], and then refined into flammable and explosive, vulnerable population, crowded, key protection, general fire protection, and emergency shelter categories [24]. The specific fire risk factors included in each category are listed in Table 1.

To render the results reasonable, it was necessary to normalize the values of different fire risk factors. Different fire risk factors have different effects on fire; therefore, different standardized equations had to be designed. Among the factors, flammable and explosive, vulnerable, crowded, key protection, and general fire protection locations are positive risk factors, while emergency shelter is a negative risk factor. Each fire risk factor influenced the comprehensive fire risk to varying degrees. An expert scoring method was used to evaluate and normalize each fire risk factor, where the weights of the six fire risk factors, including flammable and explosive, were set to 0.6, 0.4, 0.3, 0.4, 0.2, and 0.1, respectively [24]. The fire risk factors and their properties are listed in Table 2.

According to the nature of each evaluation factor, the standardized equations were determined as follows: (V represents the standardization value, X represents the independent variable, and A is the boundary value of X).

(1) $$\mathrm{Flammable}\;\mathrm{and}\;\mathrm{explosive}\;\mathrm{value}\;\mathrm{in}\;a\;\mathrm{grid}\;V=0.6\;\times \;\{1-{\left[e^{\frac{-(X+1)}{\left|A\right|}}\right]}^{5}\}\;\times \;A,$$

(2) $$\mathrm{Vulnerable}\;\mathrm{population}\;\mathrm{value}\;\mathrm{in}\;a\;\mathrm{grid}\;V=0.4\;\times \;\{1-{\left[e^{\frac{-(X+1)}{\left|A\right|}}\right]}^{5}\}\;\times \;A,$$

(3) $$\mathrm{People}\;\mathrm{crowded}\;\mathrm{value}\;\mathrm{in}\;a\;\mathrm{grid}\;V=0.4\;\times \;\{1-{\left[e^{\frac{-(X+1)}{\left|A\right|}}\right]}^{5}\}\;\times \;A,$$

(4) $$\mathrm{Key}\;\mathrm{protection}\;\mathrm{value}\;\mathrm{in}\;a\;\mathrm{grid}\;V=0.3\;\times \;\{1-{\left[e^{\frac{-(X+1)}{\left|A\right|}}\right]}^{5}\}\;\times \;A,$$

(5) $$\mathrm{General}\;\mathrm{fire}\;\mathrm{protection}\;\mathrm{value}\;\mathrm{in}\;a\;\mathrm{grid}\;V=0.2\;\times \;\{1-{\left[e^{\frac{-(X+1)}{\left|A\right|}}\right]}^{5}\}\;\times \;A,$$

(6) $$\mathrm{Emergency}\;\mathrm{shelter}\;\mathrm{value}\;\mathrm{in}\;a\;\mathrm{grid}\;V=0.1\;\times \;\{{\left[e^{\frac{-(X+1)}{\left|A\right|}}\right]}^{5}-1\}\;\times \;A.$$

#### 2.1.2. Fire Risk Quantification

The kernel density method was used to quantify the density distribution of various POI data, and the density of each POI category was used to describe the corresponding fire factor so that a value for each factor could be obtained from the grids. The improved SAVEE model was further used to quantify the spatial heterogeneity of fire risks. The basic concept of SAVEE is to select appropriate factors and perform standardized processing to obtain a value for each factor. All factor values are then added using the SAVEE formula. This method allows for complex decision-making and evaluation problems to be quantitatively processed [29]. The SAVEE model uses different calculation formulas for factors with different characteristics, thus improving on the shortcomings of traditional weighting evaluation methods.

All risk factors are standardized in SAVEE and different factors are converted into [−1,1]. The SAVEE algorithm designs different standardized formulas for different factors. The normalization equation for positive factors are as follows:

(7) $$V=\{\begin{array}{cc} 1-{\left[e^{\frac{-(X+1)}{\left|A\right|}}\right]}^{5}, & V\propto X\\ {\left[e^{\frac{-(X+1)}{\left|A\right|}}\right]}^{5}, & V\propto \frac{1}{X} \end{array},$$

and for negative factors are as follows:

(8) $$V=\{\begin{array}{cc} -{\left[e^{\frac{-(X+1)}{\left|A\right|}}\right]}^{5}, & V\propto X\\ {\left[e^{\frac{-(X+1)}{\left|A\right|}}\right]}^{5}-1, & V\propto \frac{1}{X} \end{array},$$

where *V* is the value of a factor after standardization and *A* is the boundary value of the independent variable *X*; X≤A. V∝X indicates that the independent variable *X* is positively correlated with factor value *V*. $V\propto \frac{1}{X}$ means that the independent variable *X* is negatively correlated with factor value *V*.

**Figure 1:**
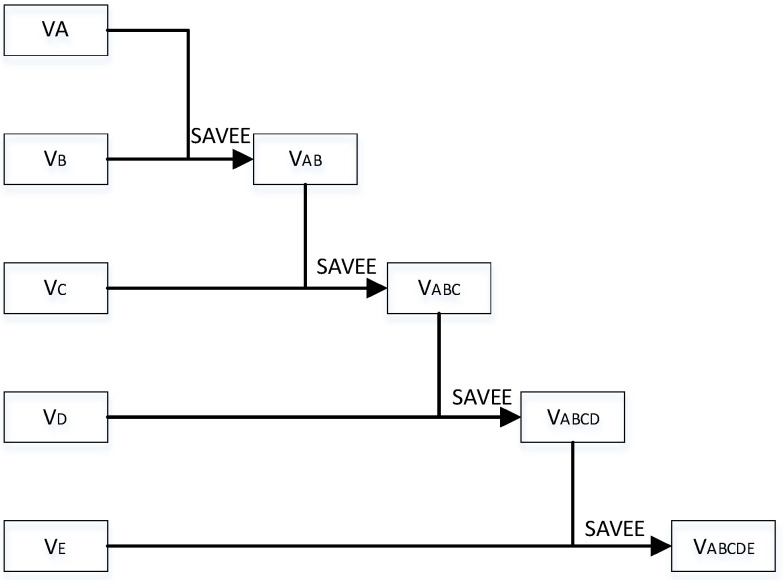
Flow of superposition equations in SAVEE calculation.

The standardized values of all factors are superimposed pair by pair to obtain comprehensive evaluation results for each grid. The equation used for summing factors is as follows:

(9) $$V_{AB}=\{\begin{array}{ll} V_{A}+V_{B}-V_{A}V_{B} & V_{A}>0,V_{B}>0\\ V_{A}+V_{B}+V_{A}V_{B} & V_{A}<0,V_{B}<0\\ \frac{V_{A}+V_{B}}{1-\min\left[\left|V_{A}\right|,\left|V_{B}\right|\right]} & \mathrm{el}se \end{array},$$

where $V_{A}$ is the standardized value of factor *A*, $V_{B}$ is the standardized value of factor B, and $V_{A}\in \left(-1,1\right)$,$V_{B}\in \left(-1,1\right)$; $V_{AB}$ is the value of factors *A* and *B* following superposition. The pairwise operation of the factors is successively repeated until all factors are involved in the operation (Figure 1).

### 2.2. Candidate Points for Urban Fire Stations under Constraint Conditions

**Figure 2:**
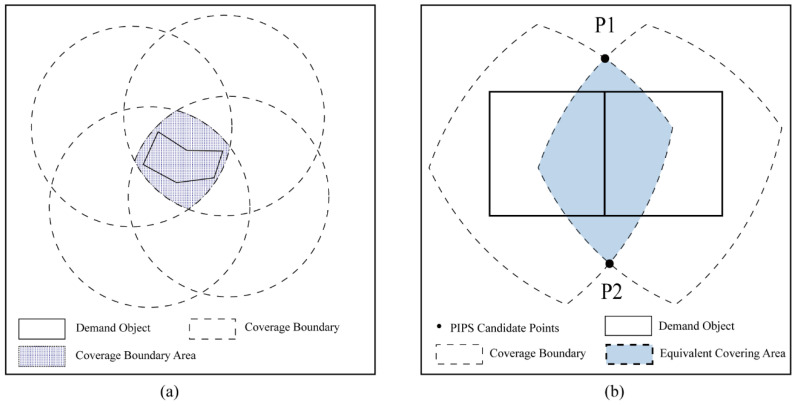
Setting the candidate points. (**a**) Determination of the covering boundary for the polygon requirement object. (**b**) Candidate selection by polygon intersection point set (PIPS).

In this study, the polygon intersection point set (PIPS) [30] was introduced to obtain candidate points in the research area. PIPS ensures that at least one optimal candidate point can cover a demand area. Moreover, it can transform an infinite set of candidate points into a finite set of points. Thus, the spatial continuous location problem can be simplified into a binary problem for calculating candidate points. A binary coverage model (complete coverage or no coverage) was therefore adopted to calculate the candidate points. The coverage range of each candidate point has a certain limit in terms of distance, but the candidate point should completely cover the corresponding demand area (a polygon). The regional boundary that completely covers an area of demand is called the coverage boundary (Figure 2a). Adjacent demand objects may have overlapping coverage boundaries; in this study, the overlapping area was defined as the equivalent covering area. Any point within the equivalent coverage area should completely cover two adjacent demand objects. Setting candidate points in such a region can improve the coverage efficiency and narrow the scope of the solution [30]. Therefore, to ensure that high-risk areas are covered by more fire stations, the candidate points for PIPS were set in the equivalent coverage area (Figure 2b).

### 2.3. Multi-Objective Optimization Based on Backup Coverage

The obtained fire station candidates had to meet the constraints of the specific spatial location requirements for the fire station. For example, a fire department should be able to reach an area for which it is responsible within 5 min of receiving an alarm. Therefore, fire stations should be located adjacent to streets such as intersections to ensure that vehicles can quickly move. The distribution of urban fire stations should follow the urban fire planning specifications, which are used to restrict the selection of fire station candidate points. For example, according to the Fire Protection Law in the People’s Republic of China (2019 Amendment), Urban Fire Prevention Planning Standards (2015), and the Wuhan Master Plan (2010–2020), the floor area of ordinary fire stations is approximately 3300–4800 m^2^, and the jurisdiction of a fire station should not be greater than 7 km^2^. Therefore, the candidate points that are generated based on PIPS should be further screened to satisfy these spatial constraints. Because the scope and capacity of each fire station are limited, the coverage of a fire station should be maximized within a reasonable range. Traditional coverage models, when optimizing resource allocation, try to cover as large a demand area as possible under the limited number of facilities; while most of them ignore the fire risk in the demand area. However, in areas with relatively high fire risks, there may be situations in which multiple demands for assistance simultaneously occur in different areas. It is hard to offer timely service for each demand area because of the limitation of traditional models. Therefore, it is necessary to supply backup for high fire-risk areas as much as possible.

To build fire stations that can reasonably supply backup for high fire risk areas, two types of cover are defined as follows:

Once coverage: An area of demand is covered by one fire station, the demand area is located within the service range of one fire station.

Backup coverage: An area of demand is covered by at least two fire stations; the demand area is located in the service range of more than one station. Thus, a high fire risk area can be covered by several fire stations.

The rules for selecting locations for urban fire stations can be summarized as follows: (1) the distribution of urban fire stations should be designed in accordance with urban fire planning principles; (2) the fire stations should maximize once coverage to cover more demand areas; (3) for areas with high fire risk, fire stations should utilize backup as much as possible; (4) the driving distance should be limited to a relevant time period and free-flow speed; (5) because fire risk spatially varies, areas with high fire risks should be more likely to include fire stations. Accordingly, the backup coverage model of multi-objective optimization for urban fire stations was built as follows:

I, J: the set of demand areas and potential fire stations, respectively;
i, j: the index of demand areas and potential fire stations, respectively;
a_i_: estimated fire risk in-demand area i;
d_ij_: the distance between i and j;
S: service standard;
N_i_: the set of fire stations capable of suitably serving demand i, $N_{i}{=\{j|d}_{\mathrm{ij}}\leq S\}$;
p: the number of fire stations points that qualify;

The decision variables:

$$y_{i}\{\begin{array}{cc} 1 & \mathrm{demand}\;\mathrm{area}\;i\;\mathrm{is}\;\mathrm{provided}\;\mathrm{with}\;\mathrm{once}\;\mathrm{coverage}\;\mathrm{service}\\ 0 & \mathrm{otherwise} \end{array}$$

$$r_{i}\{\begin{array}{cc} 1 & \mathrm{demand}\;\mathrm{area}\;i\;\mathrm{is}\;\mathrm{provided}\;\mathrm{with}\;\mathrm{once}\;\mathrm{coverage}\;\mathrm{service}\\ 0 & \mathrm{otherwise} \end{array}$$

$$x_{j}\{\begin{array}{cc} 1 & \mathrm{if}\;a\;\mathrm{fire}\;\mathrm{station}\;\mathrm{is}\;\mathrm{sited}\;\mathrm{at}\;j\\ 0 & \mathrm{otherwise} \end{array}$$

(10) $$\mathrm{Max}\;Z_{1}=\sum_{i\in I}a_{i}y_{i},$$

(11) $$\mathrm{Max}\;Z_{2}=\sum_{i\in I}a_{i}r_{i},$$

s.t

(12) $$r_{i}+y_{i}\leq \sum_{j\in N_{i}}x_{j}\;\forall i\in I,$$

(13) $$r_{i}\leq y_{i}\;\;\;\;\forall i\in I,$$

(14) $$\sum_{j\in J}x_{j}=p,$$

(15) $$x_{j},r_{i},y_{i}\in \left\{0,1\right\}\forall j\in J,\forall i\in I,$$

Objective function (10) maximizes once coverage, and is designed to ensure that the location optimization of fire stations effectively minimizes costs. Objective function (11) maximizes the number of fire stations that are available for service in high fire risk areas. Constraint (12) indicates that the sum of the coverage times accepted by the demand site must be greater than or equal to the sum of once coverage and backup protection coverage. Constraint (13) indicates that if the demand site accepts backup coverage, it must also receive once coverage. Constraint (14) indicates that the number of facilities is limited to p. Constraint (915) indicates that $x_{j}$ and $r_{i}$ are binary variables with a value of 0 or 1. The most favorable locations for fire stations $y_{i}$ can be obtained by optimizing this model.

In order to simplify the calculation process, a single objective equation is obtained by using linear weighting to merge the two objective functions of the multi-objective equation.

The final single objective equation is shown below:

(16) $$\mathrm{Max}\;Z=\sum_{i\in I}a_{i}y_{i}+\sum_{i\in I}a_{i}r_{i}$$

where *a_i_* is the estimated fire risk in-demand area *i*; other parameters have the same meaning as the multi-objective equation above.

## 3. Results

### 3.1. Study Area

**Figure 3:**
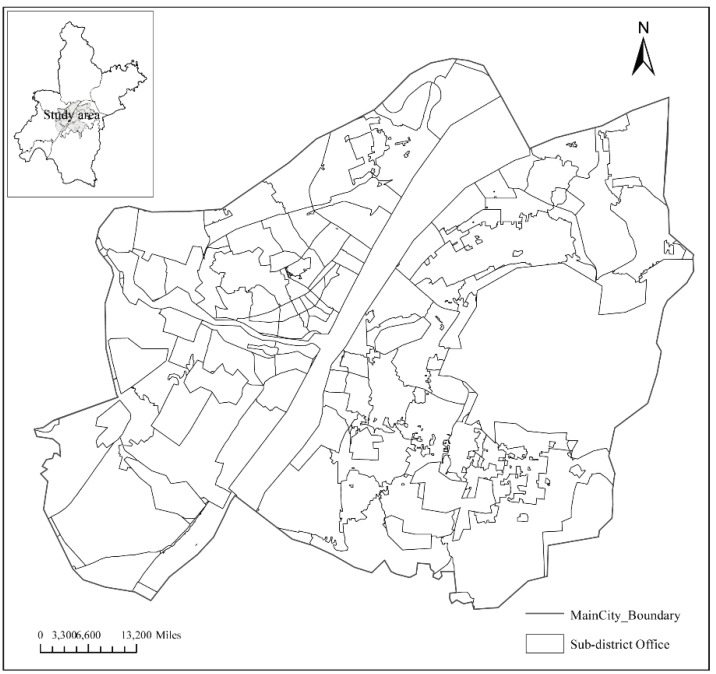
Study area in Wuhan.

Wuhan City was considered as the study area. (Figure 3). Wuhan, the capital of Hubei Province, is located in the middle reaches of the Yangtze River. The main urban area of Wuhan City covers a total area of 678 km^2^. The city has experienced rapid urbanization over the past few decades. Experimental results based on the urban land use planning map of Wuhan City was used to validate the proposed models and verify the applicability of the optimal location model of fire stations.

Road network: The road network data describing the main urban area in Wuhan were downloaded from the Open Street Map website and used to compute the driving time from the fire stations to each fire point. Different levels of road networks were used in the study, which included motorways and primary, secondary, tertiary, and trunk roads.

POI data: The POI data in the main urban area of Wuhan includes flammable and explosive, vulnerable population, crowded venues, key protection, general fire protection, and emergency shelter categories. Flammable and explosive POIs include gas stations, LPG stations, and factories. POIs in the vulnerable population category include general hospitals and schools. Crowded POIs include business areas such as shopping malls, supermarkets, and entertainment venues and facilities associated with transportation, such as subways and train stations. Key protection POIs include government offices, scenic spots, and scientific, educational, and cultural services, such as libraries, science and technology museums, archives, art galleries, and museums. General fire category POIs mainly refer to residential areas and emergency shelter category POIs mainly refer to emergency shelters. A total of 29,570 POIs were collected, which had four attributes, including name, address, longitude, and latitude.

Policy constraints and other data: According to the *Urban Fire Prevention Planning Standards (2015)*, the jurisdiction of fire stations should not exceed 7 km^2^. The “2019 Q1 Traffic Analysis Report of Major Cities in China” released by AutoNavi Maps states that the free flow speed in Wuhan is 47.71 km/h. The speed of a fire engine is determined according to the free velocity. The time taken for a fire squadron to receive instructions and prepare for dispatch is 1 min, with 4 min allocated to travel. Therefore, the distance from the fire point to station should be within 3.18 km.

### 3.2. Calculating Candidate Locations for Fire Stations under Multiple Constraints

**Figure 4:**
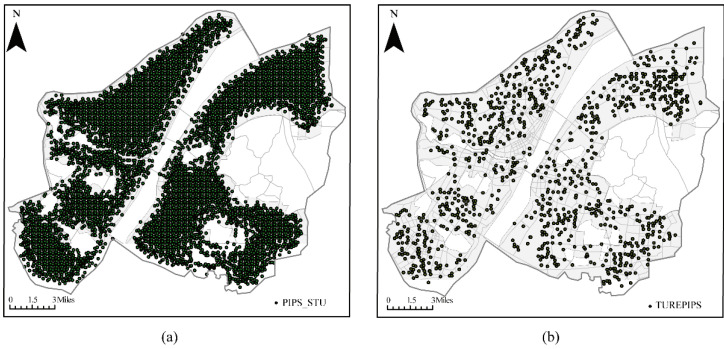
Candidate locations. (**a**) Locations determined by PIPS; (**b**) candidate locations including spatial constraints.

The concept of PIPS was utilized to calculate the candidate locations for a fire station. However, to comply with urban fire protection planning regulations, the candidate locations determined by the PIPS must meet the spatial distribution characteristics of fire stations. For example, the fire station should be located in an area with several adjacent streets to ensure that the fire engine can quickly move. Inflammable and explosive chemical points need to be covered by a fire station within 200 m, and the distance between fire stations and public buildings with large populations, such as hospitals, schools, kindergartens, nurseries, theaters, or shopping malls, should be not less than 50 m. Therefore, the locations of fire stations should further constrain these spatial distribution characteristics to avoid inappropriate candidate locations. Subsequently, network analysis was used to achieve the constraints of the above spatial characteristics. A total of 6259 locations were obtained with PIPs (Figure 4a); however, only 1432 locations were retained after these spatial constraints were included (Figure 4b).

### 3.3. Regional Fire Risk Assessment

**Figure 5:**
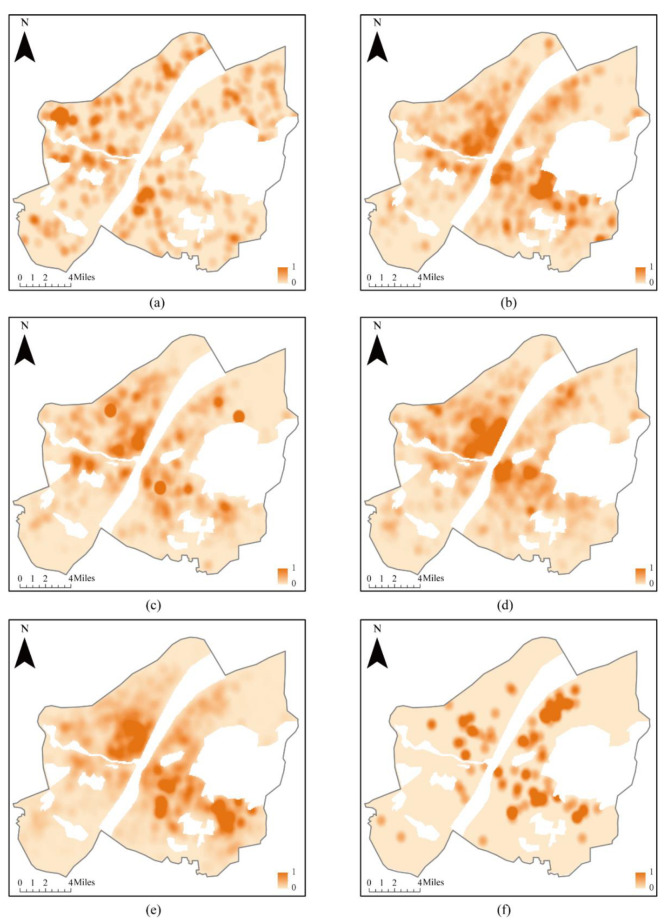
Kernel density distribution diagram of various fire risk factors: (**a**) flammable and explosive; (**b**) vulnerable population; (**c**) crowded population; (**d**) key protection; (**e**) general fire; (**f**) emergency risk aversion.

Based on previous research [30] in which fire risk factors were classified, POIs were reclassified into six fire risk factors. Of the 29,570 POIs in the study area, 543 were flammable and explosive, 1734 included vulnerable populations, 3212 were crowded population, 7273 were areas allocated key protection, 16,676 were general firefighting, and 132 were emergency risk aversion types. The spatial distribution of fire risk that was obtained using point data for various types of fire risk factors is shown in Figure 5.

**Figure 6:**
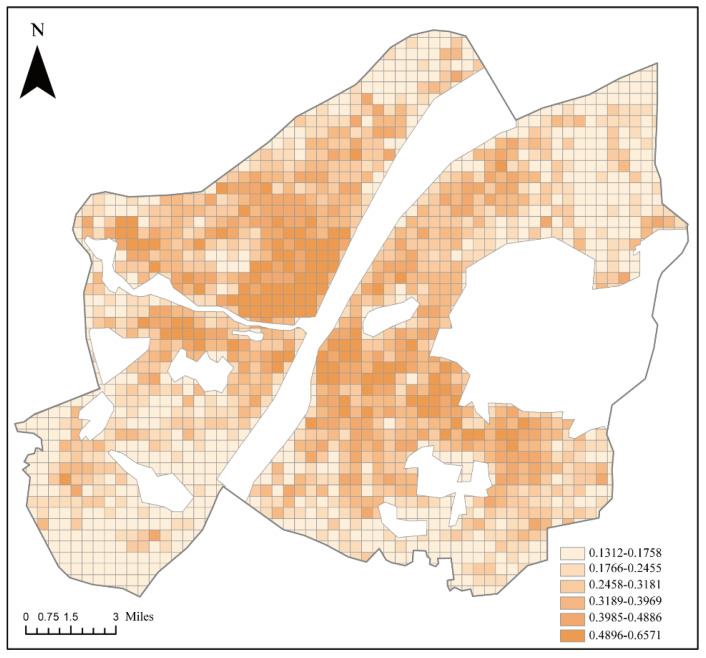
Spatial distribution of fire risk in central Wuhan.

According to the standard formulas mentioned in Section 2.1.1, the comprehensive fire risk value was determined by the SAVEE model using the standardized values for different types of fire risk factors (Figure 6).

### 3.4. Selection and Coverage of Fire Stations Based on Backup Coverage

The weight of each grid reflects the coverage priority of each requirement object, which is an important parameter in the multi-objective optimization model. In this study, general coverage, backup coverage, and risk coverage rates were introduced to evaluate the performance of fire station location. The general coverage rate (*r_c_*) is defined as the ratio of demand areas that are covered by fire stations in the study area, which can be determined as follows:

(17) $$r_{c}=\frac{\sum_{i\in I}y_{i}}{I},$$

where *I* represents the collection of demand areas. $y_{i}$ equals 1 when demand area *i* is provided with once coverage service by the corresponding fire station; otherwise, it is 0. The backup coverage rate (*r_Bc_*) is defined as the ratio of demand areas that are covered by two or more fire stations, which can be defined as follows:

(18) $$r_{Bc}=\frac{\sum_{i\in I}r_{i}}{I},$$

where *I* represents the collection of demand areas. $r_{i}$ equals 1 when demand area *i* is serviced by two or more fire stations; otherwise, it is 0. The risk coverage rate (*r_Rc_*) is defined as the ratio of the total risk in the demand area covered by fire station services to the total risk, which is defined as given below:

(19) $$r_{Rc}=\frac{\sum_{i\in I}a_{i}y_{i}}{\sum_{i\in I}a_{i}},$$

where *I* represents the collection of demand areas and $a_{i}$ represents the demand weight of demand area *i*. $y_{i}$ equals 1 when demand area *i* is provided with once coverage service by the corresponding fire station; otherwise, it is 0.

**Figure 7:**
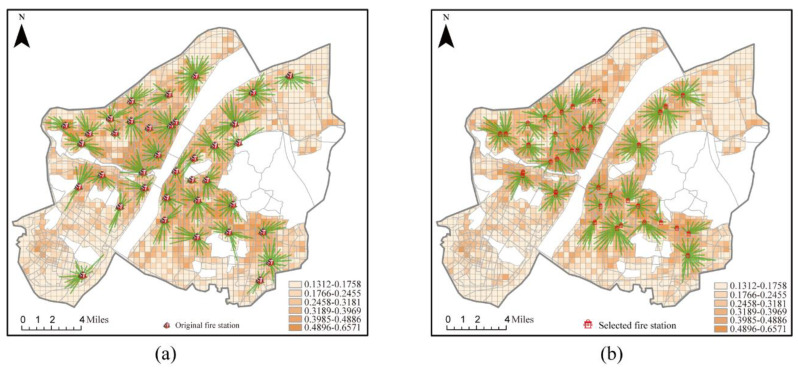
The performance and optimization of existing fire stations in terms of location obtained by the proposed method (*p* = 37). (**a**) Performances of the existing fire stations; (**b**) performances with the backup coverage model.

A total of 37 fire stations have been built in Wuhan so far, and the study area was divided into 1504 demand areas (Figure 7a). The 37 existing fire stations can cover 705 demand areas with a coverage rate of 46.88% and a risk coverage rate of 57.26%. A total of 75 demand areas were covered by more than one fire station and had a backup coverage rate of 4.99%. Using the proposed model, the distribution locations of the same number of stations could be optimized (Figure 7b). Thirty-seven fire stations could cover 621 demand areas with a coverage rate of 41.29% and a risk coverage rate of 54.00%. A total of 257 demand areas were covered by more than one fire station, with a backup coverage rate of 17.09%. Compared with the performance of the existing fire stations, the backup coverage rate of the fire stations selected using the proposed method was improved by 6.86%.

**Figure 8:**
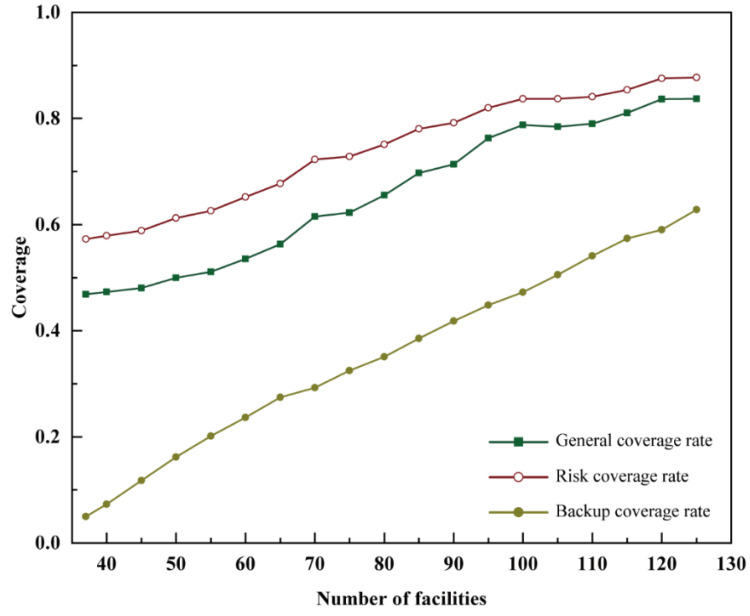
Number of facilities and coverage using the backup coverage model (BCM).

IBM CPLEX Optimization Studio was used to calculate the specific spatial distribution of a total of 40 to 125 facilities, including the 37 existing fire stations, in five-step increments using the backup coverage model (Figure 8). According to the impact the number of facilities had on the coverage relationship, based on the backup coverage model, it was apparent that the backup coverage rate significantly improved as the number of fire stations increased. When the total number of fire stations in an area was less than 100, increasing their number significantly improved the general coverage rate and risk coverage rate. This effect was less significant when there were more than 100 fire stations; although the backup coverage rate increased with the increase in the number of fire stations, the rate of general coverage and risk coverage did not significantly improve. A total of 40 fire stations could cover 712 demand areas with a general coverage rate of 47.34%. The total risk in the study area was 402.73, and 233.19 of the value at risk could be covered, with a risk coverage rate of 57.9%. At the same time, 110 demand areas were covered by more than one fire station with a backup coverage rate of 7.31%. When the total number of fire stations was increased to 105, the backup coverage exceeded 50%. Meanwhile, most of the high fire risk areas were covered by fire stations. The results suggest that the proposed backup coverage model can solve the problem of high fire risk areas requiring more coverage.

## 4. Discussion

### 4.1. Fire Station Optimization Considering Existing Stations

**Figure 9:**
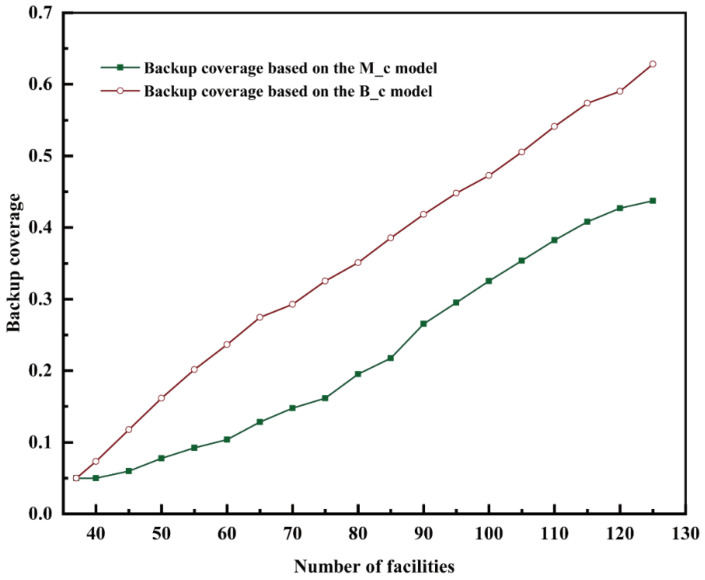
Comparison of backup coverage in the two models.

When selecting locations for building new fire stations in a city, the existing fire stations are crucial constraints. In this study, the addition of fire stations was designed, taking into consideration the existing fire stations. The performance of the maximal coverage model and the backup coverage model were analyzed using the same number of facilities (Figure 9). It was apparent that the backup coverage rate when using the backup coverage model was always greater than when using the maximal coverage model, particularly when increasing the number of fire stations. The proposed backup coverage model considered both the backup coverage and maximum coverage, while the maximum coverage model only considered once coverage. When the total number of fire stations was less than 65, the difference between the backup coverage rate in the two models was observed to continually increase. However, the backup coverage rate was still better under the backup coverage model (the backup coverage rate always remained higher than 14.5% compared with the maximum coverage model). However, the backup coverage rates of the two models were not saturated, indicating that backup coverage will continue to increase when the number of fire stations is increased under both models. The difference in backup coverage between the two models was the largest (19.08%) under 125 fire stations.

**Figure 10:**
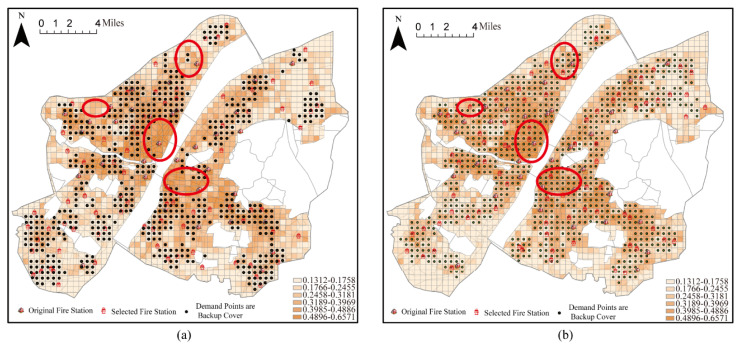
Coverage of fire stations considering existing stations. (**a**) Location optimization by the MCM; (**b**) location optimization by BCM.

When the number of fire stations was set to 115 (including the 37 existing fire stations) using the maximal coverage model, a demand area of 99.54% was covered by one fire station and 40.82% of the area was covered by more than one fire station. Therefore, the demand area was almost completely covered. However, the backup coverage for high-risk areas was still insufficient (Figure 10a). For example, high-risk areas (red circle in Figure 10) should contain more fire stations when backup coverage is not available. At this point, 44.29% of the fire risk in the study area was under backup coverage. However, when the number of fire stations was set to 115 (including the 37 existing fire stations) using the backup coverage model, 85.40% of the demand areas were covered, and 57.38% of the demand areas were covered by more than one fire station (Figure 10b). Almost all of the demand areas with backup coverage were located in areas with high fire risk, and the total risk of the area covered by backup coverage was 278.27, with backup coverage of 69.10%. Under the same conditions, the total fire risk in the demand area covered by the backup increased by 24.81%. This result reflected the enhancement in the coverage of higher fire risk areas under the backup coverage model.

### 4.2. Fire Station Optimization without Considering Existing Stations

**Figure 11:**
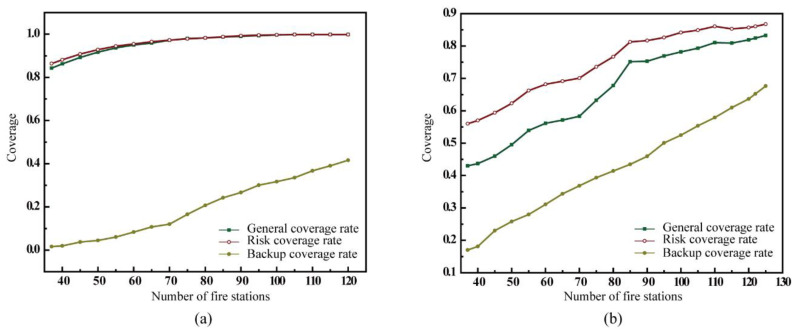
Number of facilities and coverage rate. (**a**) Performance in maximal coverage model (MCM); (**b**) performance in backup coverage model (BCM).

When planning the locations of fire stations in a new city, few fire stations may be built. In this study, we analyzed the difference between the maximal coverage model and proposed backup coverage model without considering the existing fire stations. The changes in several coverage rates were analyzed in accordance with the number of fire stations based on the maximal coverage model (Figure 11a). The results show that the risk coverage rate was always higher than the backup coverage rate; however, the difference between the risk coverage rate and the general coverage rate was minimal. This is because the maximum coverage model prioritized serving areas with high fire risk only once and did not improve the coverage for high fire risk areas with more fire stations. Therefore, in the same situation, the risk coverage rate was the highest among the three coverage rates.

When the general coverage rate is very high (the number of facilities is more than 40 and the general coverage rate is greater than 85%), it is difficult to close the gap with the general coverage rate because there is scope for improvement. As the number of fire stations increased, all three types of coverage rates increased, with the backup coverage rate increasing the most rapidly. Furthermore, the backup coverage rate increased at a faster rate after the number of facilities reached 70. However, the general coverage rate and risk coverage rate were much more extensive than the backup coverage rate. When 70 fire stations were present, the general coverage rate and risk coverage rate exceed 97%, rendering it difficult to improve the coverage rate. However, the back coverage rate was only 11.97%, and there was still scope for improvement. Therefore, although the maximum coverage model did not consider back coverage as the optimization goal as the number of fire stations increased, the corresponding back coverage rate could be improved.

When the total number of fire stations was the same as that of the existing fire stations, 37 fire stations, the risk coverage rate exceeded 85%. Obviously, the maximal coverage model intentionally reduced the coverage in the same demand area to achieve a greater coverage effect. However, when the maximal coverage model was utilized, the backup coverage rate was less than 10% until the number of fire stations reached 65. In this situation, some high fire risk areas could not be backup covered, leading to failure in response to situations where more than one demand point was present or several high fire risk areas were covered by the same fire station.

When the backup coverage model was adopted, the general coverage rate, risk coverage rate, and backup coverage rate increased as the number of facilities increased. The results (Figure 11b) indicate that the risk coverage rate was consistently higher than the general coverage rate and backup coverage rate. Moreover, with the increase in the number of fire stations, all three types of coverage increased. This shows that the backup coverage model can improve the backup coverage of the demand areas. With a total of 95 fire stations, backup coverage could be provided to more than half of the demand areas, improving security in the higher-risk areas. With 115 fire stations, the general coverage rate and risk coverage rate remained the same, while the backup coverage rate continued to increase. This result further confirmed the purpose of proposed model as improving the backup coverage in high-risk areas.

**Figure 12:**
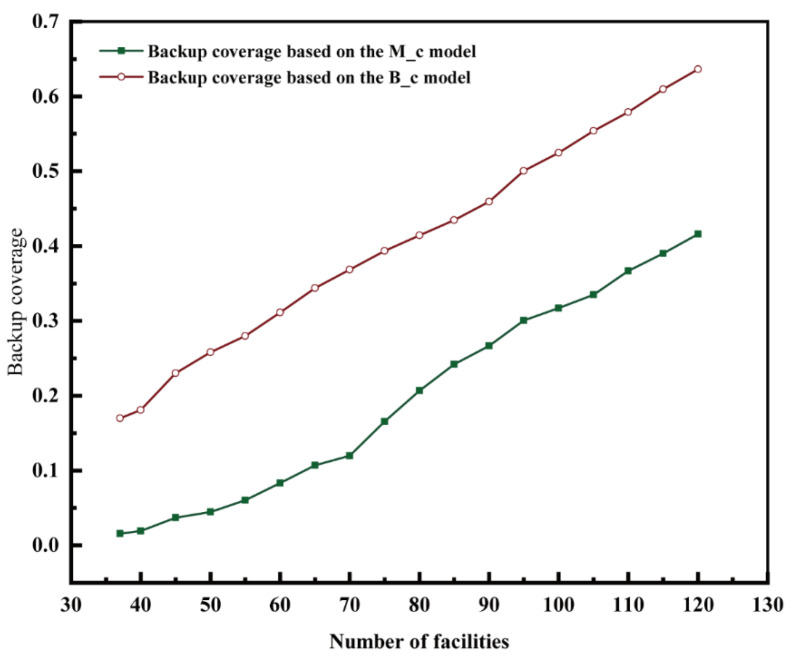
Comparison of backup coverage in the two models (MCM, BCM).

Comparing the backup coverage rate in the maximal coverage model and the proposed backup coverage model with the same number of facilities, the backup coverage model always performed better (Figure 12). The difference in performance between the two coverage models was the largest when 70 facilities were included, amounting to 24.87% (11.97% for the maximal coverage model and 36.84% for the backup coverage model). However, the general coverage rate and risk coverage rate obtained based on the backup coverage model were less than 85%. Therefore, even if the gap in the backup coverage between the two models was the largest, it was not a safe choice to choose a layout with 70 fire stations in the study area. When the number of facilities was set at between 70 and 90, the rate at which the growth of the backup coverage rate in the maximal coverage model increased. When the number of facilities exceeded 90, the backup coverage rate based on the maximal coverage model declined, whereas the backup coverage rate based on the backup coverage model improved. When 115 facilities were present, the risk coverage rate based on the backup coverage model was >85% and the backup coverage rate was >60%, which covered the risky areas in the study area. Therefore, it is possible to enhance the coverage for high-risk areas in the study area if 115 facilities are available.

**Figure 13:**
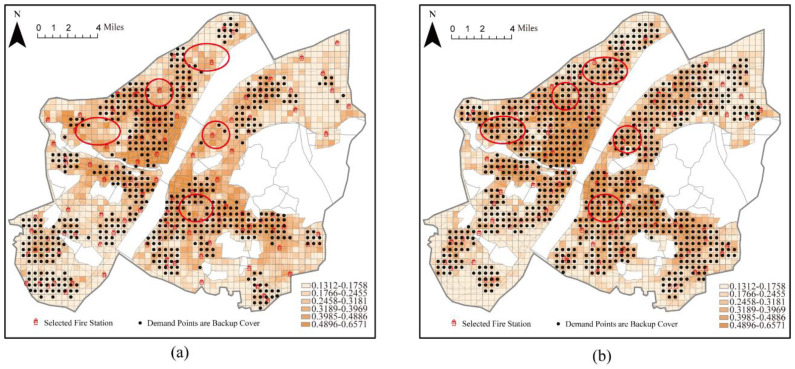
Coverage under different models. (**a**) Maximal coverage model; (**b**) backup coverage model.

For further analysis, the number of fire stations was set to 115 (without the 37 existing fire stations) in both the maximal coverage model and the proposed backup coverage model. In the maximal coverage model, the backup coverage rate reached approximately 40%. At this point, the total risk of the area covered by the backup was 180.12 and the risk coverage rate was 44.72%. Many areas without backup coverage were located in areas with higher fire risk, indicating that the locations selected by the maximal coverage model did not reinforce areas with high fire risk (Figure 13a). However, >60% of the demand areas were covered by more than one fire station under the backup coverage model. These areas with backup coverage are located in areas with high fire risk. At this point, the total risk of the area covered by backup was 290.30, accounting for 72.08% of the fire risk in the study area. The proposed backup coverage model aimed to backup coverage in areas with high fire risk, thus enabling these regions to have more fire station coverage (Figure 13b). For example, more than one demand point in the same high fire risk area or more than one high fire risk area are covered by the same fire station.

## 5. Conclusions

Effective fire control deployment and timely fire rescue can significantly reduce the risks associated with fire. In this study, we proposed a multi-objective optimization method, known as the backup covering model, to select the optimal locations for fire stations. The method combined the construction rules of urban fire stations with urban road networks and the cost constraints of driving. Several types of POI were used to quantify the fire heterogeneity of the region in order to prioritize areas with high fire risk. The proposed model was adopted to optimize the location of fire stations in the main urban area of Wuhan. Experiments showed that the proposed backup coverage model could enhance the safety of high fire risk areas by providing higher backup coverage in areas with high fire risk demand. Moreover, the designed model can make sure the limited number of fire stations can cover as more places as possible. These results suggest that the number of fire stations can be gradually increased to provide residents with more scientifically reliable and effective fire protection strategies. Additionally, the results of this study may be used to assist government decision-makers in making more informed decisions when designing the location of fire stations according to the spatial heterogeneity of fire risk. Inevitably, because of the preference for services in high fire risk areas, the model ignores services in low fire risk areas when there are not enough fire stations. To estimate the fire risk more reasonably, in future research, historical fire data and POI data could be integrated to consider the spatial heterogeneity of fire risk more comprehensively. Moreover, fewer fire stations will be a constraint to optimate the proposed.

## Figures and Tables

**Table 1:** Classification of fire risk factors.

Fire Risk Factor	The POI Types
flammable and explosive	gas stations, LPG stations, and factories
vulnerable population	general hospitals and school
crowded	shopping malls, supermarkets, entertainment venues, subways, and train stations
key protection	government offices, scenic spots, scientific premises, libraries, science and technology museums, archives, art galleries, and museums
general fire protection	residential areas
emergency shelter	emergency shelters

**Table 2:** Fire risk factors and properties.

Serial	Evaluation Factor	Positive Factor	Negative Factor	Weight
1	flammable and explosive	√		0.6
2	population vulnerable	√		0.4
3	crowded population	√		0.4
4	key protection	√		0.3
5	general fire	√		0.2
6	emergency risk aversion		√	0.1

√ means the factor belongs to corresponding positive or negative factor.

## Data Availability

Not available.
